# Development and Validation of a Personalized Survival Prediction Model for Uterine Adenosarcoma: A Population-Based Deep Learning Study

**DOI:** 10.3389/fonc.2020.623818

**Published:** 2021-02-18

**Authors:** Wenjie Qu, Qingqing Liu, Xinlin Jiao, Teng Zhang, Bingyu Wang, Ningfeng Li, Taotao Dong, Baoxia Cui

**Affiliations:** ^1^ Cheeloo College of Medicine, Shandong University, Jinan, China; ^2^ Department of Obstetrics and Gynecology, Qilu Hospital of Shandong University, Jinan, China

**Keywords:** adenosarcoma, survival prediction, deep learning, artificial intelligence, database, personalized model

## Abstract

**Background:**

The aim was to develop a personalized survival prediction deep learning model for adenosarcoma patients using the surveillance, epidemiology and end results (SEER) database.

**Methods:**

A total of 797 uterine adenosarcoma patients were enrolled in this study. Duplicated and useless variables were excluded, and 15 variables were selected for further analyses, including age, grade, positive lymph nodes or not, marital status, race, tumor extension, stage, and surgery or not. We created our deep survival learning (DSL) model to manipulate the data, which was randomly split into a training set (n = 519, 65%), validation set (n = 143, 18%) and testing set (n = 143, 18%). The Cox proportional hazard (CPH) model was also included comparatively. Finally, personalized survival curves were plotted for randomly selected patients.

**Results:**

The c-index for the CPH model was 0.726, and the Brier score was 0.17. For our deep survival learning model, we achieved a c-index of 0.774 and a Brier score of 0.14 in the external testing set. In addition, the limitations of the traditional staging system were revealed, and a personalized survival prediction system based on our risk scoring grouping was developed.

**Conclusions:**

Our study developed a deep neural network model for adenosarcoma. The performance of this model was superior to that of the traditional Cox proportional hazard model. In addition, a personalized survival prediction system was developed based on our deep survival learning model, which provided more accurate prognostic information for adenosarcoma patients.

## Introduction

Adenosarcoma is a rare tumor of the female genital tract, accounting for approximately 5% of uterine sarcoma. The National Comprehensive Cancer Network (NCCN) and International Federation of Gynecology and Obstetrics (FIGO) have published clinical practice guidelines and staging systems for adenosarcoma. Standard treatment is total hysterectomy with bilateral salpingo-oophorectomy, and neither radiotherapy nor chemotherapy has been proven beneficial (1). Patients with stage I disease often have a good prognosis with a 5-year survival of 60–80% (1). However, tumors demonstrate sarcomatous overgrowth (>25% of the total tumor volume consists of pure sarcoma) course with a higher rate of recurrence and death (2). Due to the low incidence and histological diversity of uterine adenosarcoma, only a few case reports and series provide data on prognostic factors and survival prediction (3). Researchers have developed prognostication studies with different methods, including univariate analysis, multivariate analysis, multivariable Cox regression and the Kaplan-Meier method (4, 5), among which the most commonly used is multivariable Cox regression analyses. However, whether these traditional methods accurately work remains debatable. Therefore, an accurate prognostication system is crying needed for treatment decisions and survival prediction.

As mentioned above, most researchers were restricted to the low incidence and rare cases of adenosarcoma. The surveillance, epidemiology and end results (SEER) database is a population-based data source covering approximately 34.65% of the U.S. population. Clinical data have been collected since 1973, including the stage of cancer, histopathological subtypes, treatment modality, and survival data (6). The database has been used in various studies to perform survival analyses of all malignant tumors, including adenosarcoma (7). However, most of these studies used the Cox proportional hazard (CPH) model, which cannot handle nonlinear correlations in survival analyses.

With the rapid development of artificial intelligence, a new choice is provided for adenosarcoma researchers. The deep learning method allows a machine to be fed with raw data and to automatically discover the representations needed for detection with the use of multiple neural layers in the network (8). It has the ability to analyze the nonlinear correlations that are more common in the real world. It has been proven to be greatly effective for various clinical tasks, including image identification (9, 10), pathological diagnoses (11–13), genomic analysis (14, 15), metabolomics (16), and immunology (17) studies. Combining the SEER database with the deep learning method is a good choice that has been proven to be valuable in many cancer prognosis studies, such as breast cancer and lung cancer (18, 19). However, studies taking advantage of the abundant cases in the SEER database and the high efficiency of the deep learning method are absent in the prognosis of adenosarcoma.

In this study, we aimed to develop a survival prediction deep learning model for adenosarcoma patients collected from the SEER database. With this model, better prediction was achieved. We also attempted to develop a new personalized survival prediction system based on the model we established.

## Materials and Methods

### Data Collection

The SEER Program is a comprehensive source of population-based information in the United States that includes the cancer stage at the time of diagnosis and patient survival data. It updates annually and is provided as a public service for researchers.

The SEER database had 133 usable variables. In this study, we used “CS Schema v2040+”, which was collected under the specifications of a particular schema based on site and histology, to select corpus adenosarcoma patients from 1973 to 2014. Only cases with one primary tumor were included in our study. We also excluded cases with incomplete follow-up data. Cases with a follow-up time equal to 0 which might indicate death in the hospital or other recording error were excluded too because of their great uncertainty. We kept corpus adenosarcoma patients of all stages, and the final sample size was 797.

Since the SEER dataset utilized publicly available desensitized data, this study did not need approval from the institutional review board (IRB) or informed consent from patients.

### Data Preparation

Among 133 original variables, we excluded those duplicated variables using correlation matrix analyses. The selected variables for further analyses were age, year when patients were diagnosed, diameter of tumor, grade, Hispanic status, number of excised lymph nodes, positive lymph nodes or not, number of positive lymph nodes, metastasis, marital status, race, extension of tumor, stage, surgery or not, and surgery type.

Stages were defined from the farthest extension of the tumor and lymph nodes involved as category variables. The SEER catalog is named the Extent of Disease (EOD), which is used for cases diagnosed before 2004, and Collaborative Stage (CS), which is used for data after 2004. We also redefined marital status as single, married, divorced and windowed. In the SEER database, several methods were introduced to define race. In this study, we classified race into white, black, and Asian, among which Hispanic was singled out as two classified variables. In addition, two classified variables also included positive lymph nodes or not and surgery or not. Grade was defined as a category variable indicating undifferentiation and low, moderate or high differentiation. Moreover, surgery type was defined as a category variable indicating local excision, hysterectomy and bilateral adnexectomy plus or not plus lymphadenectomy. The number of excised lymph nodes, number of positive lymph nodes, diameter of tumor, and age were defined as continuous variables. Extension of the tumor, which indicated localization, parametrium or distance, was defined as a category variable.

### Deep Survival Neural Network

The original multitask logistic regression model was first developed by Chun-Nam Yu in “Deep neural networks for survival analysis based on multitask framework” (20). Then, S. Fotso updated this model to a kind of neural multitask logistic regression model (N-MTLR) in “Learning patient-specific cancer survival distributions as a sequence of dependent regressors” (21). In this work, we used this kind of N-MTLR to develop our deep survival neural network for survival analyses.

In summary, this model first transformed patient follow-up time into a series of time vectors annotated with 0. If a patient had the event (=1), then the corresponding time point changed to 1. For a censored patient, all of the censored time vectors were annotated as 1.

### Statistical Analyses and Evaluation of Models

Overall survival was defined as the final outcome of patients, which was measured by interval time between diagnoses and death or loss of follow-up. Both the CPH model and deep survival learning model were evaluated in this study. For the deep learning model, we used the independent testing set to evaluate the performance to prevent potential overfitting. We used the concordance index (c-index) and the integrated Brier scores (IBS) to evaluate the performances of different models. Differences between predicted and actual data were also recorded.

Kaplan-Meier curves and Cox regression analyses for patients staged with the traditional staging system were performed. The difference was considered significant if the P value was less than 0.05. Our model assigned precise weights to each variable after data training and multiple iterations. According to the final weights, a risk score was calculated by the DSL model. we developed new staging groups according to the risk score. Finally, personalized survival curves were also plotted for randomly selected patients from the testing set.

The deep learning model was developed on the PyTorch framework. Scikit-learn and pandas packages were also used for the treatment of data. We also used STATA software (version 13) for other statistical analyses.

## Results

### Patient Demographics and Characteristics

**Figure 1:**
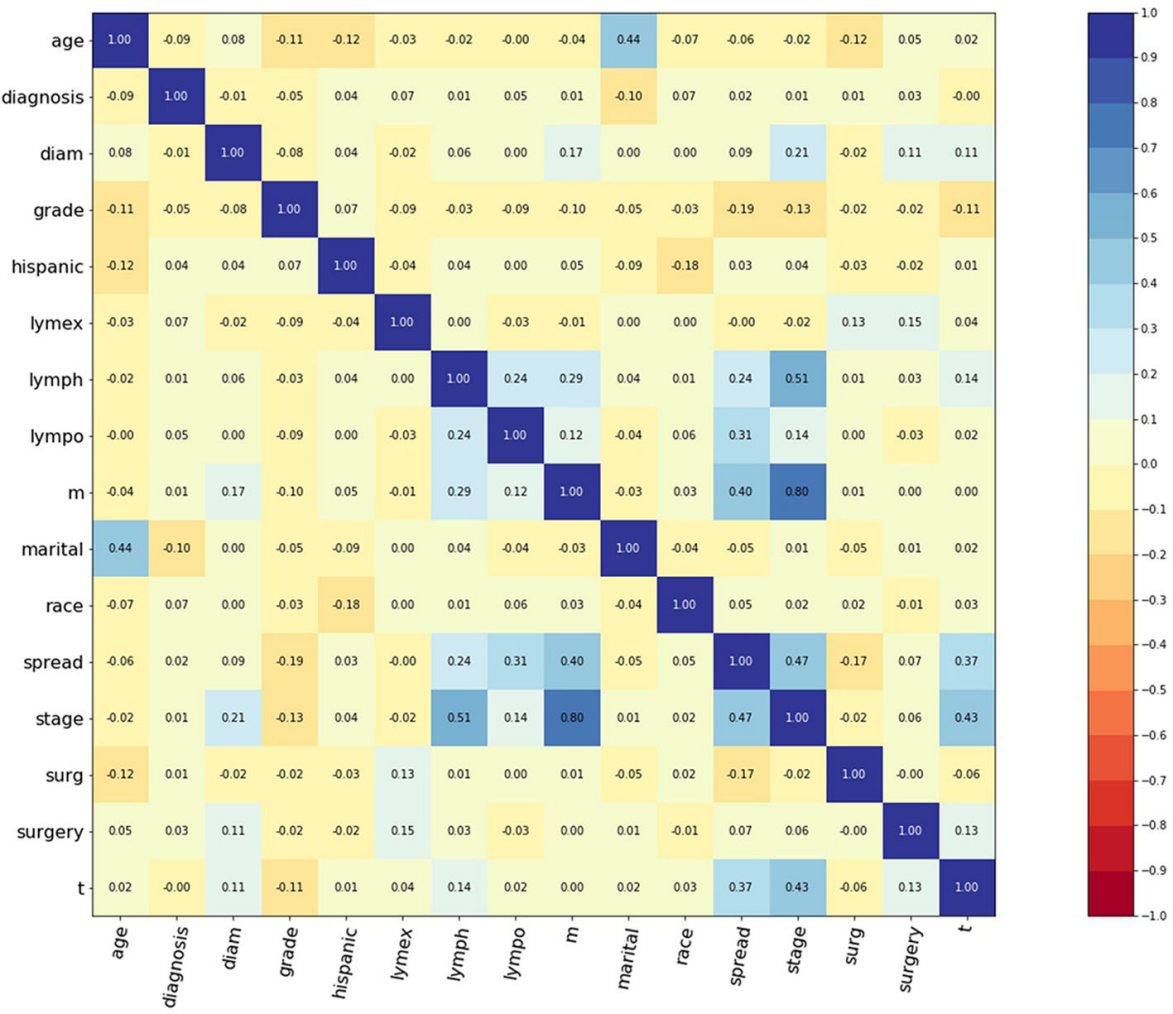
Correlation matrix of 15 selected features. Values in this figure indicated the correlation coefficient of two corresponding variables. Color indicated strength of correlation, in which dark blue indicated strong positive, and dark red indicated strong negative relationships. Diagnosis: year when patients were diagnosed. Diam: diameter of tumor. Lymex: number of excised lymph nodes. Lympo: number of positive lymph nodes. Lymph: positive lymph nodes or not. Spread: extension of tumor. Surg: surgery or not. Surgery: surgery type.

A total of 797 corpus adenosarcoma patients registered from 1973 to 2014 in the SEER database were enrolled in this study. A correlation matrix was plotted, and 15 variables correlated with survival were selected (**Figure 1**). The selected data were randomly and automatically split into a training set (n = 519, 65%), validation set (n = 143, 18%) and testing set (n = 143, 18%).

The patient demographic characteristics are shown in **Table 1**. A total of 594 cases were white (75.8%), 115 were black (14.7%), and 75 were Asian (9.5%). A total of 172 cases were single (22.9%), 388 were married (51.7%), 85 were divorced (11.3%), and 106 were widowed (14.1%). Eighty-eight cases were undifferentiated (24.7%), 52 were poorly differentiated (14.6%), 133 were moderately differentiated (37.2%), and 84 were highly differentiated (23.5%). A total of 588 patients had localized tumors (77.7%), 127 patients extended to the parametrium (16.8%), and 42 patients extended to a distance (5.5%). A total of 239 cases were stage I (88.1%), 11 were stage II (4.1%), 7 were stage III (2.6%), and 14 were stage IV (5.2%). Twelve patients underwent local excision surgery (11.2%), 56 underwent a hysterectomy and bilateral adnexectomy (52.3%), and 39 underwent a hysterectomy and bilateral adnexectomy plus lymphadenectomy (36.5%).

**Table 1:** Patients demographic and clinicopathological characteristics.

	Characteristics	No. (%)
Age, years (n = 797)	Mean SD	56.8 14.8
Race (n = 784)	White Black Asian	594 (75.8%) 115 (14.7%) 75 (9.5%)
Marital status (n = 751)	Single Married Divorced Widowed	172 (22.9%) 388 (51.7%) 85 (11.3%) 106 (14.1%)
Diameter of tumor (n = 170)	Mean SD	61.7 46.4
Grade (n = 357)	Undifferentiation Low Middle High	88 (24.7%) 52 (14.6%) 133 (37.2%) 84 (23.5%)
Number of excised lymph nodes (n = 741)	Mean SD	7.5 11.1
Number of positive lymph nodes (n = 385)	Mean SD	0.1 0.6
Extension of tumor (n = 757)	Localized Parametrium Distant	588 (77.7%) 127 (16.8%) 42 (5.5%)
Stage (n = 271)	I II III IV	239 (88.1%) 11 (4.1%) 7 (2.6%) 14 (5.2%)
Surgery type (n = 107)	Local excision TH+BSO TH+BSO+LND	12 (11.2%) 56 (52.3%) 39 (36.5%)

### Cox Proportional Hazard Model

The cox proportional hazard (CPH) model was first developed for multivariable analysis, dealing with both category and continuous variables. The concordance index (c-index) has been widely used in the survival analysis of several cancers (22, 23). Generally, when the c-index is close to 1, the model has almost perfect predicted ability, but when it is close to 0.5, the model has no power to discriminate a risk factor. In this study, the CPH model achieved a c-index of 0.726 in survival prediction.

**Figure 2:**
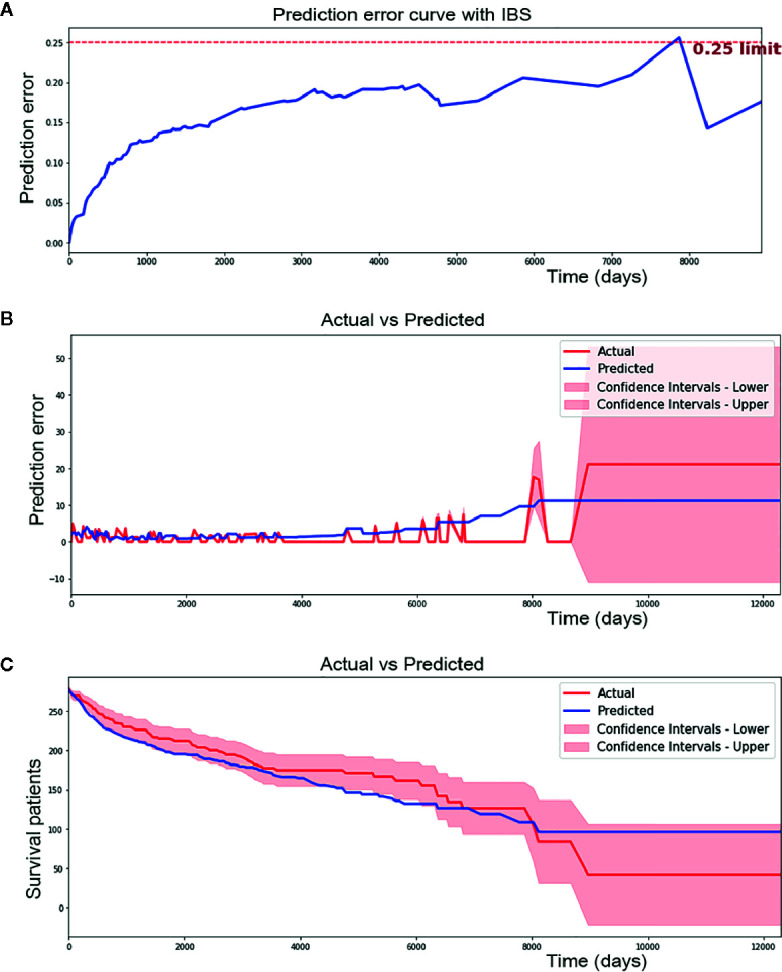
Performance of cox proportional hazard (CPH) model. **(A)** CPH model has 0.17 of IBS (below 0.25 obviously). **(B)** CPH model make a median absolute error of 1.615 patients and mean absolute error of 2.223 patients during 12000 days of follow-up time in testing set. **(C)** Predicted and actual survival curves plotted by CPH model. It made a median absolute error of 13.726 and mean absolute error of 14.626. As we can see from the figure, some spots were plotted outside the confidence intervals.

The Brier score measures the accuracy of probabilistic predictions. Because it is a cost function, a lower score indicates more accurate predictions, while a higher score indicates less accurate predictions. In this study, a Integrated Brier Score (IBS) of 0.17 was achieved using the CPH model (**Figure 2A**).

In addition, median absolute error and mean absolute error measure the variability between prediction and reality. A median absolute error of 1.615 and a mean absolute error of 2.223 were achieved by the CPH model in our study (**Figure 2B**). However, in regard to survival curves, many areas of the predicted survival curves were plotted outside the confidence intervals of actual survival curves, and greater absolute errors were shown (**Figure 2C**). The ability to perform the CPH model may be limited by missing data for many patients.

### Deep Survival Learning (DSL) Model Building

The CPH model performed well in linear relationships, while the survival problem contained nonlinear relationships in the real world. Therefore, a neural network was introduced for nonlinear relationships, while the classic deep learning method failed in handling time-to-event data. Here, we used a “multitask logistic regression model” to handle specific survival data and undertake censored data.

**Figure 3:**
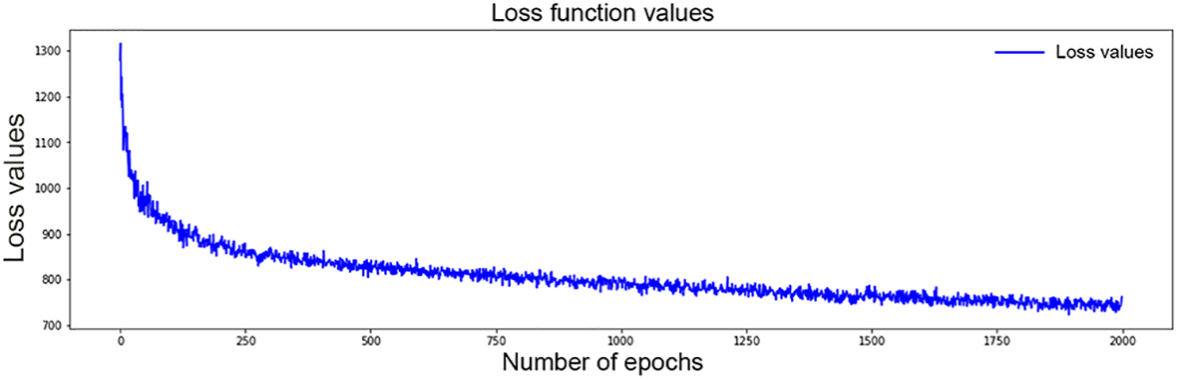
Values of loss function for DSL model decrease from 1,300 to 762 after 2,000 time of iterations.

The structure of the final model is four layers, each of which has 50 neurons. The grid search method was used for hyperparameter selection. The selected optimal hyperparameters were as follows: initial method was glorot_uniform, the dropout rate was 0.3, l2 regularization was 1e-2, l2 smooth was 1e-2, the optimizer was Adam, and learning_rate was 1e-4. After 2,000 iterations, the loss value decreased from 1,300 to 762 (**Figure 3**). Finally, a c-index of 0.831 was achieved in the validation set.

### DSL Model in the Testing Set

**Figure 4:**
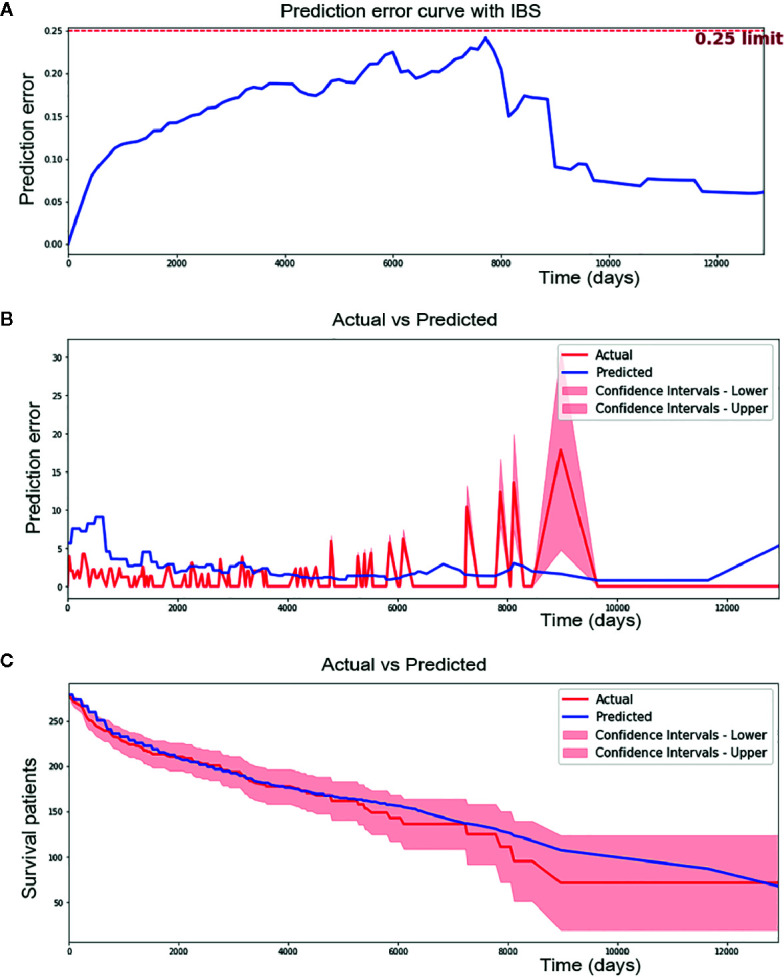
Performance of Deep survival learning (DSL) model. **(A)** In the independent testing set, we achieved 0.14 of brier score using our DSL model. **(B)** DSL model made a median absolute error of 1.989 patients and mean absolute error of 2.621 patients during 12,000 day of follow-up time in testing set. **(C)** DSL model made a median absolute error of 3.851 and mean absolute error of 5.632 in survival curve prediction. Nearly all spots of predicted curve lied within confidence intervals of actual curve and the predicted curve was drew similarly to the actual one.

Overfitting is a common prediction error in machine learning. It means a model doing much better on the training set than on the test set. In another word, a model has low generalization. To prevent potential overfitting of our model, we evaluated the performance of the DSL model using an independent testing set instead of a training set and validation set. Finally, our model reached a c-index of 0.774 and a IBS of 0.14 in the external testing set (**Figure 4A**). Moreover, 1.989 of the median absolute error and 2.621 of the mean absolute error (**Figure 4B**) were achieved in each time interval. This result suggested that our model could perform well in survival prediction.

In addition, calibration survival curves were also drawn using the testing set. Calibration curves showed that nearly all areas of the predicted survival curves were plotted within confidence intervals of actual survival curves (**Figure 4C**), suggesting that the predicted survival result was amply credible and obviously better than the CPH model in this study.

### Personalized Survival Prediction Using the DSL Model

**Figure 5:**
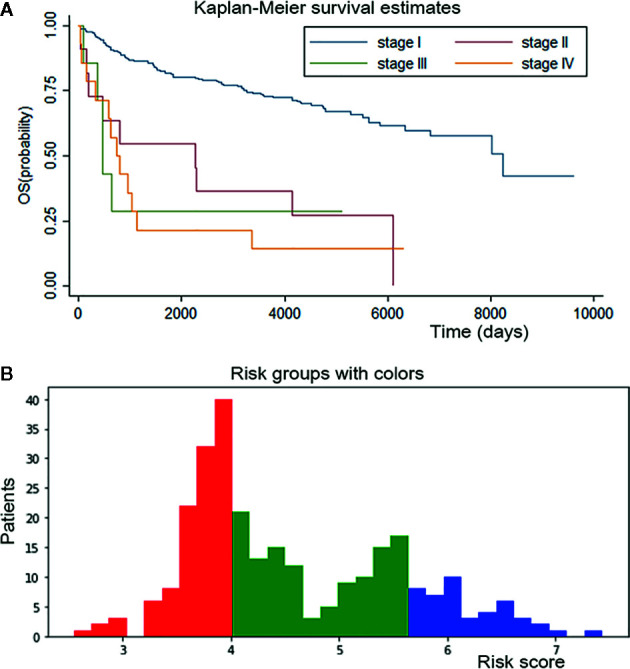
Survival curves for conventional staging system and personalized survival prediction established by DSL model. **(A)** K-M curve of conventional staging system showed significant difference between stage I from other three stages and inapparent difference between stage II, III and IV. **(B)** We divided adenosarcoma patients into three stages according to risk factors calculated by our DSL model. Patients with a score of 0-4 were classified in stage I and marked in red color, patients with a score of 4-5.5 in stage II and green color, patients with 5.5-8 score in stage III and blue color.

Kaplan-Meier curves were plotted for patients from the conventional staging system (**Figure 5A**). The difference in survival between stage I patients from the other three stages was significant (P < 0.001); however, the difference between stages II, III and IV was inapparent. Mortality for stage II, III and IV patients increased 3.9-, 4.7-, and 5.5-fold, respectively, relative to the stage I patients in Cox regression analyses.

**Figure 6:**
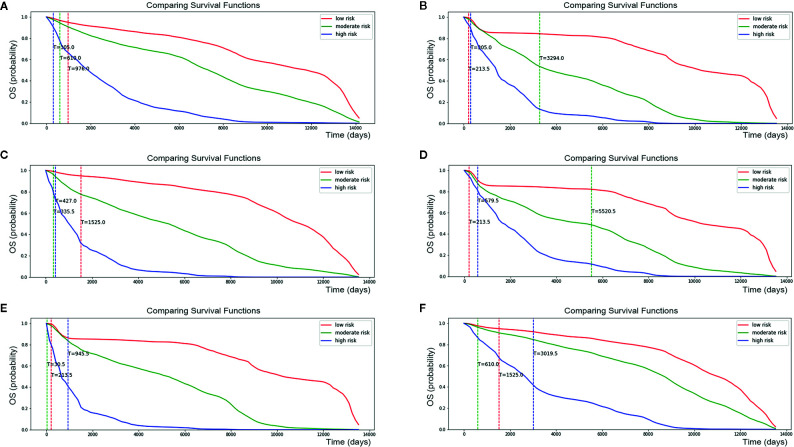
Personalized survival curve for three randomly selected patients showed apparently diverse results. After six times Repeated selections and validations **(A–F)**, Patient with low risk always has the best survival result, contrasting with patient with high risk resulting in short survival time.

Risk factors for patients were computed by our DSL model. According to our model, the risk score ranged from 0 to 8. Patients were divided into three staging groups based on the number of patients in different risk levels (**Figure 5B**). Then, one patient was randomly selected from each group of our new risk-related staging system, and survival curves were painted for the three patients. Six times Repeated selections and validations were carried out (**Figure 6**). Notable differences were observed, indicating that survival results between patients of our three stages were more significantly different than those of the traditional four stages. In other words, our model may have potential implications in personalized treatment and prediction of adenosarcoma.

In addition, dividing patients into four or three stages only provided a general impression of survival prediction, since patients differed within stages. However, our model calculated the risk score of one certain patient and describe her personalized survival curve, suggesting that our model may have a latent capacity in the personalized treatment of adenosarcoma.

## Discussion

Adenosarcoma is a rare malignancy that is often associated with irregular uterine bleeding and physical complaints. Many studies have been performed for its pathologic characteristics, treatment, and prognosis factors during past decades. Researchers aimed to determine clinical risk factors associated with decreased survival, which may guide the optimal management of this rare tumor (24). Based on previous studies, the most important prognostic factors of adenosarcoma are age, sarcomatous overgrowth, myometrial and lymphovascular invasion, and lymph node involvement; moreover, heterologous stromal components and extrauterine manifestations are also associated with poor prognosis (25). Currently, an increasing number of researchers focus on the survival prediction of adenosarcoma. Among these studies, multivariable logistic regression has been widely used to identify risk factors, and the Cox proportional hazard model has been the most widely applied to predict survival outcomes. However, these traditional methods have been proven to perform worse than the new artificial intelligence method. In this study, we established a deep survival learning model for adenosarcoma patients. To our knowledge, this is the first adenosarcoma prognostication study applying a deep learning method.

Due to the rarity of adenosarcoma, only a small case series of prognosis studies have been launched before (26, 27). Many studies have taken advantage of the SEER database due to its large population-based data (28, 29). In this study, a large number of patients provided by the SEER database were analyzed, which offered statistical strength to our conclusion.

Previous investigations have explored the ability of the CPH model to predict adenosarcoma survival outcomes (7). Since adenosarcoma is associated with multidimensional factors, the conventional model, the CPH model, for example, could not recognize complex nonlinear relationships between the variables. However, the potential for deep learning models in cancer prognostication research has also been revealed by several studies. Ole-Johan Skrede et al. (30) developed a clinically useful prognostic marker for colorectal cancer using a deep learning method and evaluated it in a large, independent patient population. Charlie Saillard et al. (31) developed two deep learning algorithms for hepatocellular carcinoma, and both models had a higher discriminatory power than a score combining all baseline variables associated with survival, confirming that the deep learning method can help refine the prediction of hepatocellular carcinoma prognosis. Since adenosarcoma is associated with multidimensional factors, the conventional model, the CPH model, for example, could not recognize complex nonlinear relationships between the variables. As we can see from our data, the CPH model performed poorly in the survival curve description, and many spots of the predicted curve were plotted outside the confidence intervals. In addition, missing data have a great impact on CPH model performance. In this study, the deep learning model performed the data imputation job and showed better performance. We contributed a better c-index of 0.774 and a Brier score of 0.14, taking advantage of the DSL model.

In addition, past works have never concentrated on the adenosarcoma staging system and prognosis-related subgroups. In our work, we found that the conventional staging system made limited contributions to predicting survival results. Several studies have shown that the majority of patients (73.4–82%) are diagnosed with stage I (32–34). However, these patients have different survival outcomes. In this study, we provided a personalized survival prediction curve for randomly selected patients, which showed a more significant difference than that of the traditional staging system. Profiting from the deep learning method, more possible risk factors were considered in the survival analysis. Brandon-Luke et al. (3) found that primary site, lymph node status, surgical procedure, chemotherapy use, race, insurance status and income quartiles were not significantly associated with overall survival of adenosarcoma using the CPH model. However, in this study, the aforementioned factors were included, and correlations within those variables were considered, which accounted for a better result.

The limitations of this study include the absence of detailed pathological information, such as sarcomatous overgrowth which has been proven to have a significant impact on survival time, as well as some molecular markers such as Ki-67 and p53 or bcl-2. Secondly, peritoneal/ascitic fluid cytology is another adverse risk factor that should be considered. But limited by SEER database, this information was unavailable. Third, only overall survival but not progression-free survival was included in our study. Progression-free survival time is also an important constitution for prognosis, indicating the period from the beginning of treatment to disease progression. However, we made a breakthrough in combining continuous overall time variables with classified death or not variables as outcome indicators, which provided more specific survival predictions.

Above all, only clinical data, including demographics and therapeutic information, were included in this study. Actually, our model could incorporate different types of data, including clinical, hematological, pathological, imaging and genetic information. Comprehensive and massive data could further improve the accuracy of survival prediction of our model. Beyond that, the therapeutic value of radiotherapy and chemotherapy is still to be proven, as well as hormone therapy. Our model could also provide evidence for the feasibility of various follow-up treatments and explore the effect of these options on prognosis with detailed treatment data. Therefore, further studies including a large series with comprehensive information, detailed survival data and multiple patient sources will be needed.

Our model gives survival time predictions that are much more accurate than the traditional survival analysis model. The personalized survival prediction system based on our DSL model showed good performance as well. The extension of our new system to an online program that can update with new measures can be expected.

## Data Availability Statement

The original contributions presented in the study are included in the article/supplementary material. Further inquiries can be directed to the corresponding authors.

## Ethics Statement

Written informed consent was obtained from the individual(s), and minor(s)’ legal guardian/next of kin, for the publication of any potentially identifiable images or data included in this article.

## Author Contributions

WQ: Conceptualization, methodology, and writing (original draft). QL: Conceptualization and methodology. XJ: Visualization and writing (review and editing). TZ: Formal analysis and data curation. BW: Formal analysis and visualization. NL: Formal analysis and data curation. TD: Conceptualization, software, and supervision. BC: Project administration and funding acquisition. All authors contributed to the article and approved the submitted version.

## Funding

This study was funded by Clinical Research Center of Shandong University (No.2020SDUCRCA007) and National Key Research & Development Program of China (2016YFC1302900).

## Conflict of Interest

The authors declare that the research was conducted in the absence of any commercial or financial relationships that could be construed as a potential conflict of interest.
